# Microcirculation Perfusion Monitor on the Back of the Health Volunteers

**DOI:** 10.1155/2013/590698

**Published:** 2013-11-28

**Authors:** Yanqi Li, Xiaomei Li, Dan Zhou, Kang Wang, Yangyang Liu, Yi Guo, Shuang Qiu, Tianchen Zhai, Shuang Liu, Jingjing Liu, Dong Ming

**Affiliations:** ^1^Experimental Acupuncture Research Center of Tianjin University of Traditional Chinese Medicine, Tianjin 300193, China; ^2^Shenzhen Renren Health Management Co., LTD, Shenzhen 518000, China; ^3^The 2nd Clinical Medical College of Beijing University of Chinese Medicine, Beijing 100078, China; ^4^Neural Engineering & Rehabilitation Lab of Tianjin University, Tianjin 300072, China

## Abstract

*Objective*. To observe the dermal microcirculation blood perfusion characterization of meridians channels (acupoints). *Methods*. 20 healthy human subjects were monitored using Pericam Perfusion Speckle Imager (PSI) for the changes in dermal microcirculation blood perfusion on governor meridian and other respective dermal regions as a control. *Result*. The microcirculation blood perfusion on Governor Meridian is higher than its control area. *Conclusion*. The dermal microcirculation blood perfusion on certain parts of Governor Meridian of healthy human subjects showed specifics.

## 1. Introduction

The meridian doctrine is one of the core theories of traditional Chinese medicine. After a half century's exploring and studying, prominent achievements have been gained in the field of the meridian doctrine and the specificity of meridians has been proved from various aspects. Biological characteristics of the meridians (acupoints) were monitored physiologically and pathologically by Contemporary scholars with many methods, including LDF and infrared spectrum detection [1]. For example, under the physical condition, ATP [2, 3], the oxygen partial pressure [4–9], transcutaneous CO_2_ emission (TCE) [10–17], temperature, and the microcirculation perfusion [18–31] at the acupoints were higher than that at the nonacupoints. Meanwhile, the corresponding indexes changed after the intervention measures like acupuncture stimulation, moxibustion, and pressure. Xu et al. [24] had found out that the dermal areas and deep tissues along the governor meridian had a higher microcirculation blood perfusion than nonmeridian controlled dermal regions on the healthy adults. In addition, the usage of electroacupuncture would result in a higher microcirculation blood perfusion. However, the current researches now were mainly focused on the distinction between the acupoints and nonacupoints instead of the meridians and nonmeridians.

At present, most of the studies on the microcirculation were proceeded with the LDF. However, the LDF had its own limitations that could not achieve a large area and real time simultaneous monitoring at the same time. This study was carried on with the PeriCam PSI System (PSI). It is a blood perfusion imager based on the Laser Speckle Contrast Analysis (LASCA) technology. It is a method that visualizes tissue blood perfusion in real-time and combine dynamic response and spatial resolution in one instrument, providing both real-time graphs and video recordings of the area of interest. LASCA provides new means to study the microcirculation in ways that were not possible in the past.

Nonlinear analysis has been applied more and more in the microcirculation analysis and provided strong supports for the microcirculation researches. Several nonlinear analysis strategies, such as fractal analysis [32] and complexity analysis [33] on the microcirculation signals, provided statistical evidence for the further understanding of the microcirculation either under the physiological or pathological conditions. The energy amount by Fourier transformation, support vector machine (SVM), and the fuzzy c-means algorithm are applied in this study. All of the three methods can describe and provide ample biological information in the microcirculation characters and provide evidences from different aspects.

The purpose of this study is to monitor the blood of the microcirculation in the back with the PeriCam PSI System (PSI) in a large area and real time, analyze data by nonlinear analysis, and compare the blood features between the governor meridian and nonmeridians so as to confirm that whether there is a difference in the dermal blood perfusion along the governor meridian.

## 2. Methods

### 2.1. Ethics Statement

This study was authorized by the Institutional Review Board of Acupuncture and Moxibustion institution, Tianjin University of Traditional Chinese Medicine (TCM). Each participant approved and signed the informed consent form before the study.

### 2.2. Subjects

Our study was conducted at the experimental acupuncture research center of Tianjin University of TCM between June and September, 2012. 20 healthy participants (10 males and 10 females) aged from 24 to 28 were recruited from Tianjin University of TCM. Smokers and alcoholics, people under recent emotional stress or have taken vasoactive drugs that may affect microcirculation, and people with skin lesions such as scars and acne on the experimental region were excluded. Participants were all informed and agreed to our study procedures.

### 2.3. Methodology

#### 2.3.1. Experiment Conditions

In our study, room temperature was controlled around 26 ± 1°C, and the relative humidity was maintained at 50%–60%. No direct sunlight or obvious indoor air convection was allowed during experiment.

The PSI parameter was set as follows: sample rate of 1 frame per second and detecting distance of 20 ± 1 cm with the whole monitoring area of 14 × 14 cm^2^. The monitoring area was also called the Region of Interest (ROI); in this study, we chose T5 as the center of the ROI. Perfusion data was recorded in real time, and the perfusion unit (PU) of the ROI was calculated and finally analyzed by the PSI System.

#### 2.3.2. Location

**Figure 1:**
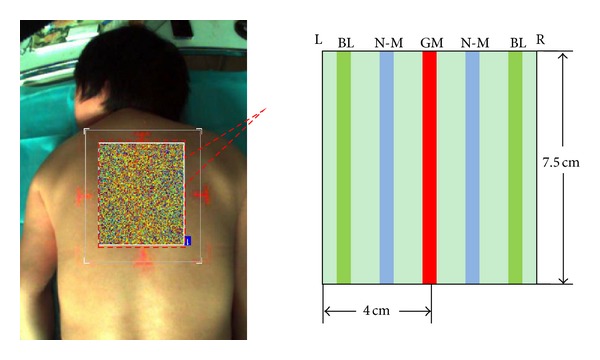
Diagram for the data acquisition.

We chose governor meridian (GM) and the two medial branches of bladder meridian (BM) as the study group; meridians were located according to the international standard. Besides, two nonmeridians (N-M), the middle line between the governor meridian, and the medial branch of bladder meridian were cited as the control group (Figure 1).

#### 2.3.3. Experiment Flow

ROI was marked and exposed to air before sampling so as to adapt to the environment; participants were in prone position and told to be relaxed. 20 min later, the blood perfusion data of the marked spot was recorded for 10 min.

#### 2.3.4. Statistics

All data was transformed, calculated, and compared using Fourier transformation, support vector machine, and fuzzy c-means algorithm.

## 3. Experiment Results

### 3.1. Experiment Subjects

20 healthy participants were included from June to September, 2012, and all of them completed the study.

### 3.2. Data Process

**Figure 2:**
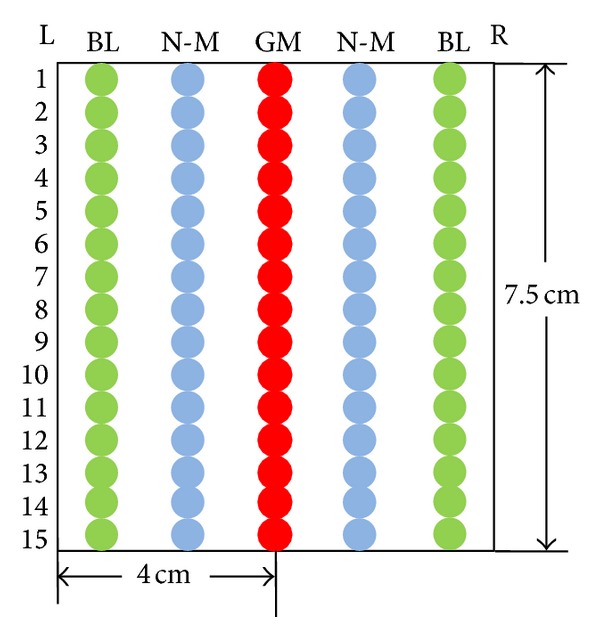
Diagram for the data acquisition.

An area of 7.5 × 8 cm^2^ of the ROI was chosen for analysis, which was basically from T2 to T9. GM, BL, and N-M (the midline of the governor meridian and bladder meridian) were marked in this filed. For further data analysis, each rectangle was separated into 15 points with 0.5 cm diameter, respectively (Figure 2).

#### 3.2.1. Energy Amount by Fourier Transformation

**Figure 3:**
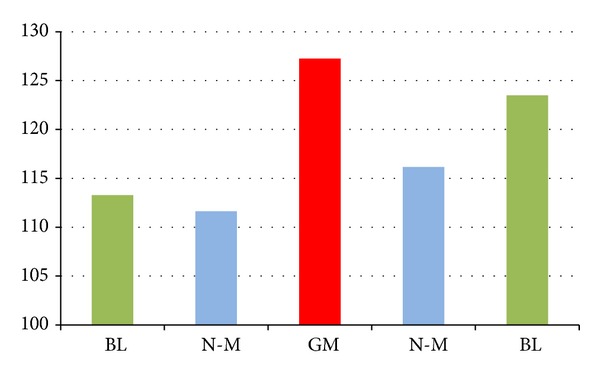
Microcirculation perfusion monitor on the back of the health volunteers. *P*_GM:  left  BL_ = 0.061; *P*_GM:  right  BL_ = 0.679; *P*_GM:  left  N-M_ = 0.014; *P*_GM:  right  N-M_ = 0.016.

Based on the theory of Fourier transformation, the acquired perfusion data was transformed into energy signals. The amount of energy of the five groups was calculated, respectively, and then compared (Figure 3).

The result of the energy amount showed that the governor meridian had the highest amount of energy, which followed by the medial branches of the bladder meridian and the nonmeridians. Moreover, the meridian and nonmeridian on the right side had higher energy than the left side. When compared statistically, there was significant statistical difference between the governor meridian and the two nonmeridians, while no statistical difference was observed between the governor meridian and the two bladder meridians or between the two bladder meridians and the nonmeridians. Therefore, the microcirculation blood perfusion on the governor meridian was obviously more activated than the other four lines.

#### 3.2.2. Support Vector Machine (SVM)

**Figure 4:**
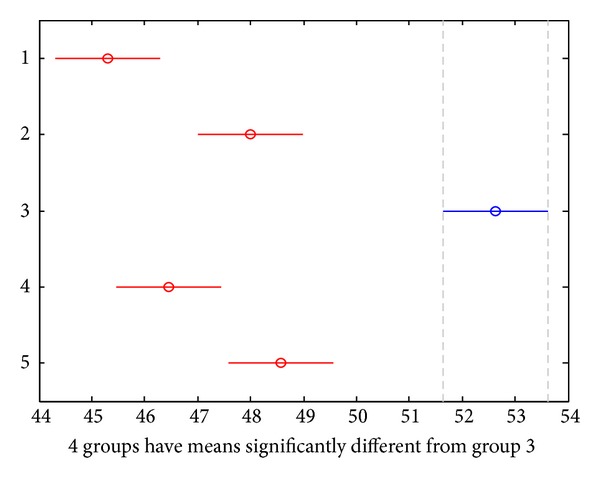
Microcirculation perfusion monitor on the back of the health volunteers; 1 denotes the left medial branch of the BL meridian; 2 denotes the left control line; 3 denotes the right control line; and 4 denotes the right medial branch of the BL meridian.

Support vector machine (SVM) is a kind of pattern recognition method based on statistical theories; it helps building a higher-dimensional space, the maximum separation hyperplane, to present all the vector data. In our study, we used this method to map the perfusion data of the five lines (governor meridian, bilateral medial branches of the bladder meridian, and the nonmeridians) into the maximum separation hyperplane. In this higher-dimensional space (Figure 4), overlapped vectors stand for no statistical difference, such as the two nonmeridians; while disoverlapped vectors imply for the existence of statistical differences, such as the governor meridian and nonmeridian. Therefore, we found out that the governor meridian had higher perfusion than either the medial branch of the bladder meridian or the nonmeridian practically and statistically.

#### 3.2.3. Fuzzy c-Means Algorithm (FCM)

**Figure 5:**
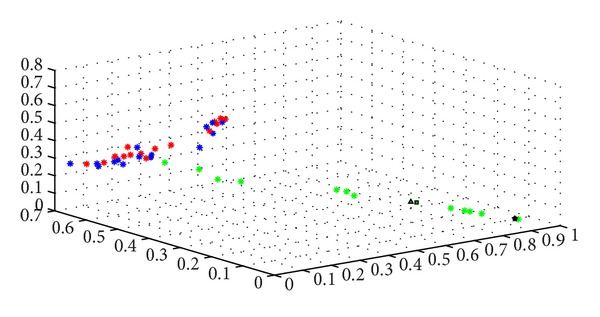
Microcirculation perfusion monitor on the back of the health volunteers. Green ones denote the governor meridian; blue ones denote the left medial branch of the BL meridian; and red ones denote the right medial branch of the BL meridian.

**Figure 6:**
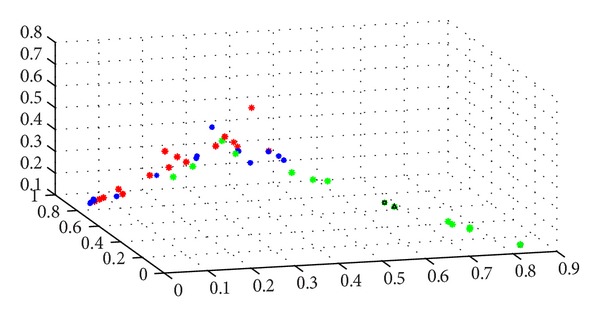
Microcirculation perfusion monitor on the back of the health volunteers. Green ones denote the governor meridian; blue ones denote the left control line; abd red ones denote the right control line.

Fuzzy c-means algorithm (FCM) was an algorithm which divides the limit numbered data into different clusters using membership function and the iterative algorithm on the premise of a defined cluster number. There were 3 clustering centers in each matrix, therefore, together *N* = 45  (15 × 3) samplings were analyzed in 20 dimensions. Perfusion data was finally output into 45 × 20 matrices, and each matrix represented the average blood perfusion of the sampling area. We mapped all the matrixes into a 20-dimensional coordinate as shown in Figures 5 and 6.

Governor meridian and the two medial branches of the bladder meridian were cited as the 3 clustering centers and samplings on governor meridian; the left and right medial branch of the bladder meridian were marked into green, blue, and red, respectively. By using FCM, a 20-dimensional chart of the 3 clustering centers was generated (as shown in Figure 5), and separated samplings in this chart stood for statistically difference of the corresponding meridians. It is clear that the PU of the green samplings (governor meridian) is significantly different from the other two colors (two bladder meridians), which means that the PU of governor meridian is statistically different from the two bladder meridians, while there is no difference between the two bladder meridians.

Use the same method to compare governor meridian with bilateral nonmeridians; similarly, mark governor meridian and the left and right nonmeridian into green, blue, and red (as shown in Figure 6). The PU of the governor meridian was significantly statistically different from the two nonmeridians, while no difference was observed between the two nonmeridians.

## 4. Discussion

### 4.1. Results

Our study showed that governor meridian had a better microcirculation blood perfusion in healthy adults and its perfusion was higher than not only the two bilateral medial bladder meridians but also the nonmeridians, which reflected the characteristics of the microcirculation of the governor meridian directly. And the results were consistent with the previous researches [23].

The microcirculation of meridians is affected by numerous factors; the permeability of capillaries as well as vasoactive agent is of great importance. During the prestage of our study, we [34] found that, by injecting EB coloring agent into the veins surrounding the ears of healthy rabbits, the EB exudation along the governor meridian on the back was higher than nonmeridian lines on the bilateral of governor meridian; it indicated that the blood vessels along the governor meridian have exceptionally high permeability. Moreover, other researchers have shown that chemicals which affect the activation of blood vessels such as CGRP, Substance P, and NO might have their own respective dispersion characteristics. For example, a study by Ma et al. [35] on the correlation between temperature changes in tissues along meridians and CGRP concentration has found that there was higher CGRP concentration in tissues along meridians with elevated temperature than in area without elevated temperatures. Cao et al. [36] have shown that electrical stimulation of “Zusanli” acupoint on rats resulted in elevated SP concentrations in the dermal layer along the stomach meridian as well as in the colon, indicating that SP is involved in both the meridian activity from acupoint stimulation and the response of the respective organ. Ma [37] has found that NO concentration is significantly higher in tissues of acupoints; histochemistry test has shown that there is elevated NO expression in nerve fibers, axons, neurons, and hair follicles of acupoints.

### 4.2. Statistical Method

In recent years, the use of bioinformatic and nonlinear dynamic analysis methods has been increasingly widespread in medical field [38–40]. For example, Li et al. [41] used bioinformatic analysis methods to illustrate the tonifying and reduction techniques from a microcirculation point of view, which effectively distinguished the difference between the lifting-thrusting reinforcing method and lifting-thrusting reducing method of acupuncture. Wang et al. [42] used system identification algorithm to obtain biological characteristics of both left and right “he gu” acupoint, demonstrating the single side nature of acupoints. In our study, we applied the nonlinear dynamic method to analyze the perfusion data. This method was also successfully used in our other researches to analyze the effect of acupuncture on nerve impulses [43] and have found that the nerve tracts discharging sequence responding to different acupuncture techniques might have respective characteristic and could be differentiated by “time span,” “frequency,” and so forth, which was an elementary step to the establishment of a scientific description of the various acupuncture techniques.

In this study, multiple nonlinear analyzing methods were applied in order to distinguish the features of the governor meridian and nonmeridian from different views and verify the specificity of the microcirculation in the physiological status, which is suitable for this study. The application of nonlinear analysis could supplement the limitations of the general medical statistical analysis and is conductive to mining more biological information, which may explain the biological meanings reflected by the experimental data.

At present, we have observed part of the characteristics of the dermal microcirculation blood perfusion of the governor meridian. The whole features of the governor meridian have not been shown. In the further research, we plan to accomplish the whole features of the governor meridian as well as other twelve regular meridians and draw the specific dermal microcirculation blood perfusion chromatogram of the fourteen meridians in order to provide evidences to the microcirculation blood changes after the interventions or under the pathological conditions.

## 5. Conclusion

Governor meridian has the feature of a high microcirculation blood perfusion in healthy people.
